# Human Extraparenchymal Neurocysticercosis: The Control of Inflammation Favors the Host…but Also the Parasite

**DOI:** 10.3389/fimmu.2018.02652

**Published:** 2018-11-16

**Authors:** Andrea Toledo, Rocio Osorio, Carlos Matus, Yazmin Martinez Lopez, Nancy Ramirez Cruz, Edda Sciutto, Gladis Fragoso, Antonio Arauz, Roger Carrillo-Mezo, Agnès Fleury

**Affiliations:** ^1^Unidad de Neuroinflamación, Instituto de Investigaciones Biomédicas, UNAM/ Facultad de Medicina UNAM / Instituto Nacional de Neurología y Neurocirugía, Ciudad de México, Mexico; ^2^Departamento de Inmunología, Instituto de Investigaciones Biomédicas, UNAM, Ciudad de México, Mexico; ^3^Clínica de Enfermedad Vascular, Instituto Nacional de Neurología y Neurocirugía, Ciudad de México, Mexico; ^4^Departamento de Neurorradiología, Instituto Nacional de Neurología y Neurocirugía, Ciudad de México, Mexico; ^5^Clínica de Neurocisticercosis, Instituto Nacional de Neurología y Neurocirugía, Ciudad de México, Mexico

**Keywords:** neurocysticercosis, *Taenia solium*, treatment, inflammation, parasite, Mexico

## Background

Neurocysticercosis (NCC) is the most common parasitic brain infection worldwide. NCC is a disease clearly associated with poverty and is endemic in most of the countries of Latin America, Africa, and Asia (1–3). Also, linked with migration, NCC is increasingly diagnosed in non-endemic countries (4, 5). In 2015, the World Health Organization identified *T. solium* as a leading cause of deaths from food-borne diseases, resulting in a considerable total of 2.8 million disability-adjusted life-years (DALYs) (6). It is caused by the establishment of the larval stage of *Taenia solium* (cysticerci) in the central nervous system. The main factors underlying the pathogeny of NCC are the location, number and stage of the parasite, the host genetic background and the host inflammatory reaction (7, 8). With respect to the latter, the intensity of the inflammation elicited by the parasite is clearly related to clinical severity (9). When parasites are located in the brain parenchyma, local inflammation promotes seizures, the most common neurological symptom in NCC patients (10). On the other hand, when parasites are located in the subarachnoid space, arachnoiditis and arteritis are frequently observed (11). Due to these potentially serious complications and to the severity of early symptoms (mainly intracranial hypertension, often requiring a ventriculoperitoneal shunt, VPS), extraparenchymal NCC (EP-NCC) is the most severe form of NCC (12).

Moreover, it should be noted that the intensity of the central inflammation is highly heterogeneous among patients, even for parasites located in the same cerebral area. Considering the cell count in the lumbar cerebrospinal fluid (CSF) as an indicator of neuroinflammation, 67% of EP-NCC patients exhibited 15–200 cell/mL; 24% presented an almost normal cell count, and around 9% of them had counts over 200 cells/mL (12). CSF protein levels are also variable in these EP-NCC patients, with 27.9% having a normal concentration (< 40 mg/dL), 60% exhibited increased levels (40–300 mg/dL), and 12.1% had very high concentrations, over 300 mg/dL (12).

## Current treatment, its objectives and pitfalls

The current treatment of NCC includes anthelminthic drugs (albendazole, ABZ, or praziquantel, PZQ) to destroy cysts, and corticosteroids (mainly dexamethasone and prednisone) to prevent complications from the exacerbated inflammatory response promoted by the parasite itself and by its destruction (13–16).

Corticosteroid treatment has been demonstrated to reduce seizure recurrence and accelerate the resolution of lesions in parenchymal NCC patients (17, 18) while reducing the frequency of VPS dysfunctions and improving the clinical outcome in patients with vasculitis associated to extraparenchymal parasites (19, 20). The dose and duration of corticosteroid treatment is practitioner-dependent, as randomized clinical trial evidences are scarce (16). However, it is generally admitted that corticosteroid treatment must be short and at doses relatively low when treating parenchymal NCC patients, and more prolonged with higher doses in cases of EP-NCC (21, 22).

The efficacy of anthelminthic therapy is variable. A clear clinic-radiological benefit is often recognized in parenchymal NCC patients, but its efficacy for EP-NCC is less immediate (11). The reasons underlying this difference are still not clear and multiple factors could be involved, like differences in parasite size, much larger in EP locations than in parenchymal ones.

## Hypothesis

We hypothesize that some central immune-inflammatory factors are required to act along with cysticidal drugs to maximize the efficiency of parasite destruction. Although other factors are also probably involved, immune-inflammatory factors could play a central role in the marked differences observed in the response to treatment among parasites located in different compartments, and in the heterogeneity of the response to treatment among patients with parasites located in the same compartment.

Indeed, parenchymal cysts are in an environment with abundance of resident immune-competent cells, while extraparenchymal ones are surrounded by CSF, a mostly acellular medium under normal conditions. Also, in swine NCC, it was demonstrated that the intensity of pericystic inflammation associated with PZQ treatment was significantly higher in parenchymal cysts than in subarachnoid ones (23). According to those reports, these differences would likely contribute to the known differences in treatment efficacy between parenchymal and subarachnoid NCC (23).

With respect to the heterogeneous response to treatment of parasites located in the same compartment, an association between the presence of proinflammatory mediators and response to treatment in EP-NCC patients was recently found (24). On the other hand, in swine NCC, ABZ alone was shown to be more effective than when combined with corticosteroids, especially in parenchymal cysts (25). Finally, in a recent study on naturally infected pigs, the administration of dexamethasone before and during PZQ treatment significantly reduced the damage to the cyst wall (26). The relevance of an exacerbated central inflammation in parasite damage might be favored by an increase in the permeability of the blood-brain barrier, favoring the influx of cysticidal drugs, as well as the arrival of peripheral inflammatory cells and mediators (27, 28).

## Implications of the hypotheses

### Neuroinflammation and its control: a double-edged sword

Medical practitioners face a dual situation, where the inflammatory reaction contributes to treatment success but where its control, necessary to elude severe neurological complications, may reduce the efficacy of the cysticidal treatment.

Currently, the administration of corticosteroids to control neuroinflammation is highly recommended (16). However, it should be noted that the use of steroids could be beneficial for the parasite in several ways. Indeed, the well-known immunosuppression promoted by steroids could favor parasite survival. Moreover, steroids stimulate the expansion of regulatory T cells and the production of TGFβ, a molecule that can also promote the survival of the cysticerci (29). An additional direct effect of corticosteroids on parasites can also play a role in the resistance to treatment. *In vitro* studies on *Taenia crassiceps* (a worm closely related to *T. solium*) showed that corticosteroids improved its ability to synthesize androgens and estrogens, enhancing its reproductive capacity (30).

### Consequences: personalized therapies are needed

**Figure 1:**
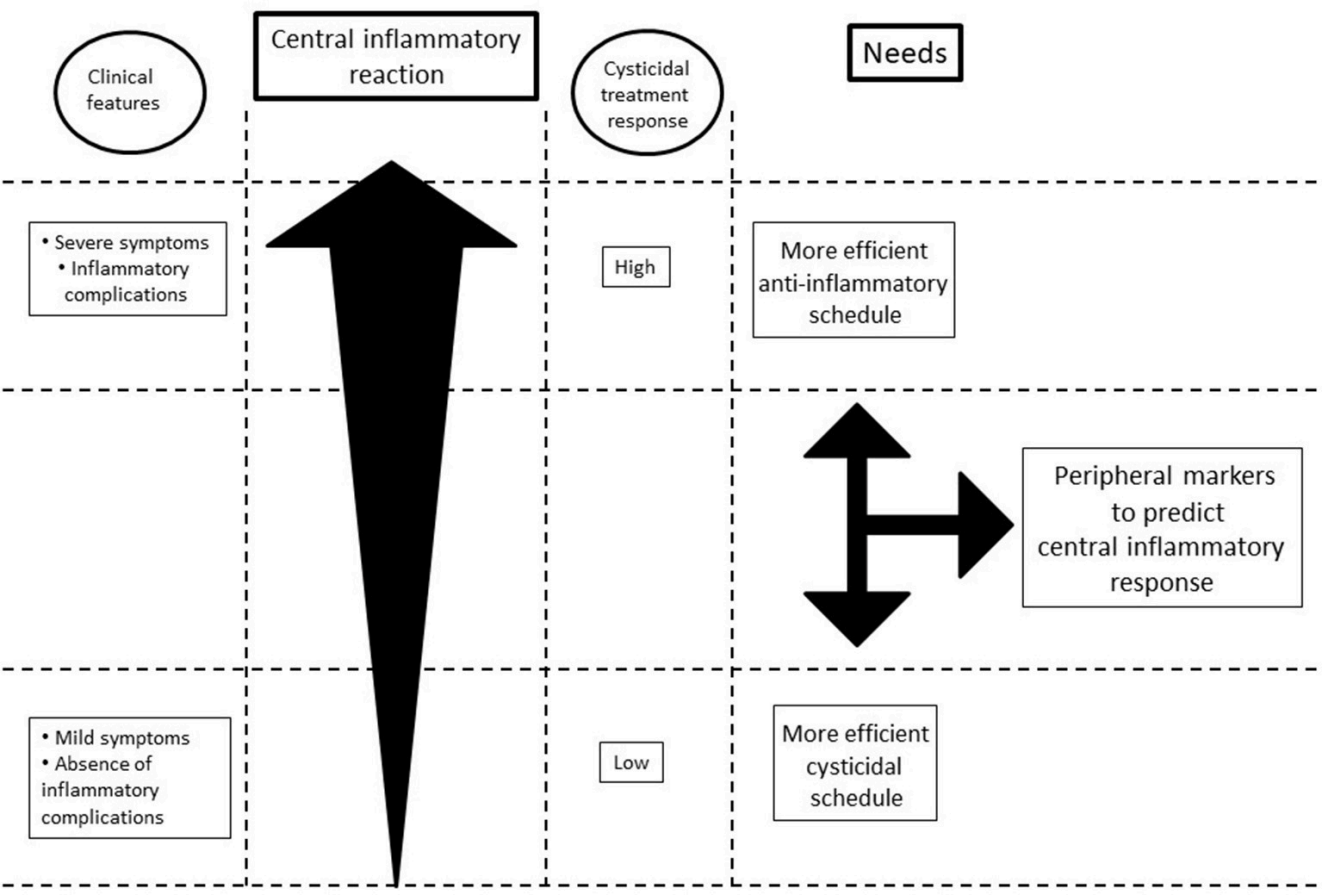
Schematic representation of the relationship among inflammation, symptomatology, and response to treatment.

This situation stressed the need for personalized therapies based on the intensity of the extremely heterogeneous central inflammatory reaction (Figure 1).

Therefore, future research on the treatment of EP-NCC should consider the following issues: With respect to anti-inflammatory drugs, the patients who exhibited less inflammation could receive lower doses without risk of inflammatory complications, thus preserving the effectivity of cysticidal drugs. On the other hand, those patients who exhibited a higher inflammatory response would require either higher doses or more efficient/specific anti-inflammatory drugs to prevent complications.

With respect to cysticidal drugs, the patients who exhibited less inflammation should receive the therapeutic schedule of 30 mg/kg/day of ABZ for 10 days (31), with the possibility of extending or repeating the treatment in case of no-response. A combined ABZ-PZQ treatment has proved to be more efficient than single drug administration in patients with more than 2 parenchymal parasites (32), and this could be an option for patients lodging extraparenchymal parasites. Research on other cysticidal drugs should continue. On the other hand, patients with a higher inflammatory status will probably respond to the current ABZ treatment schedule (15 mg/kg/day for 10 days).

## Conclusion: we need sensitive and specific peripheral biomarkers

The challenge now is how to determine beforehand which patients belong to which group (higher/lower inflammation), to provide the most appropriate combined treatment (cysticidal + corticosteroid). In this context, finding sensitive and specific new peripheral biomarkers with predictive capacity on the magnitude of neuroinflammation during the cysticidal response is much needed. CSF cellularity is currently used to evaluate central inflammation, but unfortunately lumbar puncture is not accepted in all settings and could be contraindicated when hydrocephalus is associated. The use of new radiological tools to identify infiltrated inflammatory cells and mediators in the periphery could also be very useful in the future (33), as well as studies allowing us to clarify the role of genetic in the variable treatment responsiveness of patients, since it is likely a factor of major relevance (34–36).

Finding peripheral biomarkers reflecting the intensity of central inflammation is a crucial point to reduce the morbidity of NCC resulting from the lack of response to treatment and the occurrence of inflammatory complications.

## Author contributions

AT, RO, CM, YM, and NR: made substantial contributions to conception and design, have been involved in drafting the manuscript, gave final approval of the version to be published, agreed to be accountable for all aspects of the work in ensuring that questions related to the accuracy or integrity of any part of the work are appropriately investigated and resolved. ES, GF, AA, RC-M, and AF: made substantial contributions to conception and design, have been involved in drafting the manuscript and revising it critically for important intellectual content, gave final approval of the version to be published, agreed to be accountable for all aspects of the work in ensuring that questions related to the accuracy or integrity of any part of the work are appropriately investigated and resolved.

### Conflict of interest statement

The authors declare that the research was conducted in the absence of any commercial or financial relationships that could be construed as a potential conflict of interest.

## Abbreviations

ABZ: albendazole
CSF: cerebrospinal fluid
EP-NCC: extraparenchymal NCC
NCC: Neurocysticercosis
PZQ: Praziquantel
VPS: ventriculoperitoneal shunt.
